# T2T *Colletotrichum lini* Genomes with Hifiasm: ONT R9 and R10 Read Processing and Assembly Guidelines for Fungi

**DOI:** 10.3390/jof12010045

**Published:** 2026-01-07

**Authors:** Elizaveta A. Ivankina, Ekaterina M. Dvorianinova, Alexander A. Arkhipov, Antoniy M. Kaplun, Tatiana A. Rozhmina, Ludmila P. Kudryavtseva, Nikolai M. Barsukov, Olesya D. Moskalenko, Fedor D. Kostromskoy, Kirill A. Klimov, Andrei A. Artamonov, Elena V. Borkhert, Daiana A. Krupskaya, Elena N. Pushkova, Nataliya V. Melnikova, Alexey A. Dmitriev

**Affiliations:** 1 Engelhardt Institute of Molecular Biology, Russian Academy of Sciences, 119991 Moscow, Russia; eugenevarenyuk@gmail.com (E.M.D.); arkhipov.aleksandr2.0@gmail.com (A.A.A.); atonkaplun@yandex.ru (A.M.K.); keepter@yandex.ru (N.M.B.); lesyamosk.bye-bye2003@yandex.ru (O.D.M.); fkostr@gmail.com (F.D.K.); kiry.klimov.231@gmail.com (K.A.K.); artamonov.contact@gmail.com (A.A.A.); sashai@inbox.ru (E.V.B.); zhernova.d@ya.ru (D.A.K.); pushkova18@gmail.com (E.N.P.); mnv-4529264@yandex.ru (N.V.M.); 2 Moscow Center for Advanced Studies, 123592 Moscow, Russia; 3 Federal Research Center for Bast Fiber Crops, 172002 Torzhok, Russia; tatyana_rozhmina@mail.ru (T.A.R.); lpkudryavtseva@icloud.com (L.P.K.); 4 I.M. Sechenov First Moscow State Medical University, 119991 Moscow, Russia; 5 Faculty of Biology, Lomonosov Moscow State University, 119234 Moscow, Russia

**Keywords:** *Colletotrichum lini*, anthracnose, flax pathogen, Nanopore, R9.4.1 and R10.4.1 flow cells, T2T genome assembly, basecalling tools, read processing

## Abstract

The assembly of telomere-to-telomere (T2T) genomes is essential for understanding genomic architecture, especially in fungal pathogens with complex karyotypes, such as *Colletotrichum lini*, causing flax anthracnose disease. This study provides optimized guidelines for the T2T genome assembly using Oxford Nanopore Technologies (ONT) R9.4.1 and R10.4.1 sequencing data processed with the Hifiasm 0.25.0 assembler (with --ont module). We analyzed ONT sequencing data for four *C. lini* strains and compared basecalling tools (Guppy and Dorado), read filtration strategies (quality thresholds Q10/Q15 and length cut-offs 5 kb/10 kb), and genome coverage levels from 5× to 160×. Our results demonstrated that Dorado-basecalled reads consistently had higher average quality, especially the R10.4.1 data, leading to improved telomere resolution and complete mitochondrial genome assembly. Moderate genome coverage (40–65×) combined with Q15 quality and 5 kb length filtration for R10.4.1 data, or Q10 and 5 kb for R9.4.1 data, produced the most contiguous and complete assemblies. Overfiltration of reads by length and quality or conversely excessive coverage (>90×) reduced assembly quality, causing fragmentation or erroneous chromosome merging. With optimized parameters of ONT R9.4.1 and R10.4.1 sequencing data preprocessing, Hifiasm efficiently generated T2T and near-T2T assemblies of *C. lini* genomes: 53.7–56.1 Mb length, 13–30 contigs, 12–13 chromosomes (including 3–12 T2T chromosomes), complete mitochondrial genome, and >98.5% BUSCO completeness. These findings provide a solid framework for ONT-based fungal genome assembly, facilitating future research on genomic variation and pathogenicity in *Colletotrichum* and related genera.

## 1. Introduction

*Colletotrichum lini* Manns et Bolley is a fungal pathogen that causes anthracnose in flax [1,2,3]. This disease leads to significant crop losses and is a major concern for flax farmers. Flax is used to produce oil and fiber. Flax fiber is utilized in the textile industry and composite production [4,5]. Linseed oil is rich in lignans and omega-3 fatty acids [6]. It is used as a dietary supplement and in the production of paints and varnishes [7,8,9,10]. Therefore, flax plays an important role in agriculture. Anthracnose is one of the most actively spreading diseases affecting flax and leads to substantial economic losses [11,12].

The study of the genomics of fungal pathogens can lead to more effective disease management. Obtaining a complete genome sequence of a phytopathogenic organism is essential for understanding the organization and diversity of its genetic material [13]. Many phytopathogenic species within the genus *Colletotrichum* contain accessory chromosomes, the presence of which may vary among different strains [14,15,16]. To investigate this phenomenon, telomere-to-telomere (T2T) genome sequences of phytopathogens are required [17]. Such genomes provide opportunities to identify conserved and variable regions of the pathogen genome, as well as to determine the chromosomal composition [18]. Moreover, whole genomes present new possibilities for taxonomy studies [19,20].

The third-generation sequencing on the Oxford Nanopore Technologies (ONT) platform enables the acquisition of long reads. This technology has undergone an upgrade to a new chemistry, which has improved sequencing accuracy and homopolymer resolution [21,22]. The increased accuracy allows for the assembly of high-quality genomes [23].

The Hifiasm tool is a widely used genome assembler, which was created for genome assembling from PacBio (Pacific Biosciences) reads. Later, the option of using ONT data as ultra-long reads (--ul) was added. After that, Hifiasm began to be referred to as the T2T assembler [24,25,26,27,28]. The latest version of Hifiasm (0.25.0) includes its own built-in ONT data corrector (--ont) and allows for the creation of continuous assemblies from ONT simplex data. Despite the fact that Hifiasm does not officially support R9.4.1 cells (it is not optimized for them), it still gives a good result using both R9.4.1 and R10.4.1 sequencing data. Previously, this process required the correction of reads using the Dorado correct module (HERRO), but the tool was officially released only for data generated from R10.4.1 flow cells [23,29]. The new version of Hifiasm (0.25.0) creates assemblies without the need for preliminary read correction.

In this research, we sequenced the genome of highly virulent *C. lini* strain #774 on the ONT platform and R10.4.1 flow cell and compared the obtained data with those for three *C. lini* strains’ genomes previously sequenced on the R9.4.1 flow cell. We conducted a comprehensive comparative analysis of read processing using two different basecalling software and a variety of read filtration combinations of read quality and minimum length. This study aimed to provide optimized guidelines for ONT R9/R10 read processing and T2T fungal genome assembling.

## 2. Materials and Methods

### 2.1. Fungal Material

*C. lini* strain #774 mycelium was grown in a tube with potato dextrose agar, 39 g/L (Condalab, Madrid, Spain).

### 2.2. DNA Extraction and Purification

For DNA extraction, we used our previously developed protocol [30,31] with some modifications [32]. We evaluated the quality and quantity of the extracted DNA with spectrophotometry (NanoDrop 2000C; Thermo Fisher Scientific, Waltham, MA, USA), fluorometry (Qubit 4.0; Thermo Fisher Scientific, Waltham, MA, USA), and agarose gel electrophoresis (1% agarose) techniques. The obtained DNA with A260/280 ~ 1.9, A260/230 ~ 2.2, and a concentration of ~400 ng/μL was used for the Oxford Nanopore Technologies (ONT) and Illumina library preparation.

### 2.3. DNA Library Preparation and Sequencing on the Oxford Nanopore Technologies and Illumina Platforms

To prepare the DNA library for sequencing on the ONT platform, the SQK-LSK114 Ligation Sequencing Kit (ONT, Oxford, UK) was used for *C. lini* strain #774. Sequencing was performed on a PromethION instrument with an R10.4.1 flow cell (ONT, Oxford, UK).

The Illumina library was prepared with the NEBNext Ultra II DNA Library Prep Kit for Illumina (New England Biolabs, Ipswich, MA, USA) according to the manufacturer’s protocol. Library quality and concentration were assessed using the Qsep1-Plus capillary electrophoresis system (Bi-Optic, New Taipei City, Taiwan) and Qubit 4.0 fluorometer (Thermo Fisher Scientific, Waltham, MA, USA), respectively. Sequencing was performed on a NovaSeq 6000 (Illumina, San Diego, CA, USA) instrument (150 + 150 b).

### 2.4. Genome Assembly and Quality Analyses

The obtained ONT reads of *C. lini* strain #774 (SRX30895616) and reads of previously sequenced *C. lini* strains #390-1, #757, and #771 (SRX21992304, SRX21992305, SRX21992306) [33] were basecalled using Guppy 6.5.7 (https://nanoporetech.com/software/other/guppy, accessed on 12 September 2025) with quality filtration threshold min_qscore = 10 and config files dna_r9.4.1_450bps_sup.cfg (*C. lini* strains #390-1, #757, and #771) and dna_r10.4_e8.1_sup.cfg (*C. lini* strain #774) and using Dorado 0.9.6 (https://github.com/nanoporetech/dorado, accessed on 12 September 2025) with config file dna_r9.4.1_e8_sup@v3.6 (*C. lini* strains #390-1, #757, #771) and Dorado 1.0.2 with config file dna_r10.4.1_e8.2_400bps_sup@v5.2.0 (*C. lini* strain #774) with quality filtration threshold min_qscore = 10. To remove adapters, Porechop 0.2.4 was used (https://github.com/rrwick/Porechop, accessed on 12 September 2025). Quality and quantity of the obtained reads were analyzed with SeqKit v2.4.0 [34]. If needed, reads were corrected using the HERRO algorithm [35] with Dorado 0.9.6 (correct script). The obtained Illumina reads of *C. lini* strain #774 were processed using Cutadapt 2.8 (-a AGATCGGAAGAG -A AGATCGGAAGAG) [36] and Trimmomatic 0.39 (PE, SLIDINGWINDOW:3:28, MINLEN:50) [37]. Illumina reads of the previously sequenced *C. lini* strains #390-1, #757, and #771 (SRX21992307, SRX21992308, SRX21992309) were trimmed according to the same scheme.

Assemblies were produced by Hifiasm 0.25.0-r726 with the --ont module for uncorrected reads and without any special modules for corrected reads [38]. The assemblies were polished with Pilon 1.24 [39] with Illumina reads. The prior alignments before polishing were produced with BWA 0.7.18-r1243 [40].

To analyze the quality of the obtained assemblies, completeness and contiguity statistics were calculated using BUSCO 5.8.0 (glomerellales_odb10) and QUAST 5.0.2 [41,42]. The following reference genome was used for QUAST reference-based statistics: *Colletotrichum higginsianum* IMI 349063 (NCBI Genome, GCA_001672515.1). Tidk 0.2.31 was used for the identification and visualization of telomeric repeats (https://github.com/tolkit/telomeric-identifier, accessed on 12 September 2025). The genome assemblies were aligned to each other using LAST 1471 (https://gitlab.com/mcfrith/last, accessed on 12 September 2025).

## 3. Results

### 3.1. Basecalling of ONT Reads

We chose four *C. lini* strains for this study: two highly virulent strains (#390-1, #774), one moderately virulent strain (#757), and one lowly virulent strain (#771). The genomes of *C. lini* strains #390-1, #757, and #771 were previously sequenced by us on the ONT platform with the R9.4.1 flow cell [33]. The highly virulent strain #774 genome was sequenced on the ONT platform with the R10.4.1 flow cell in the present study. To assess the impact of the basecalling software on the quality of reads and assemblies, we performed basecalling using two tools, Dorado and Guppy, with an average read quality filtration threshold of Q10 (min_qscore = 10). The characteristics of the obtained data after the Porechop adapter trimming are in Table 1.

According to these statistics and other research, Dorado and Guppy basecallers have different quality assessment approaches [43]. For the R9.4.1-sequenced data, the volume of the obtained reads for each strain varied by 0.3–0.5 Gb, which is 5–10× additional genome coverage. Maximum read length was also different. For the strain #390-1, the maximum read length varied from 104.8 kb (Dorado) to 377.4 kb (Guppy). We suppose that it was because of the quality assessment differences, so the longest read did not pass the quality threshold during Dorado basecalling. For all strains’ data, the Dorado-basecalled reads had a higher average read quality. However, the difference in the average read quality for the R9.4.1-sequenced data was not significant between the basecallers (variation of 0.2–0.3), while for the strain #774 (R10.4.1-sequenced data), the read quality value was 19.2 for Dorado and 17.1 for Guppy. The latest Dorado versions were created only for R10.4.1 sequencing data, so we considered their basecalling to be more accurate.

### 3.2. Processing of ONT Reads

**Figure 1:**
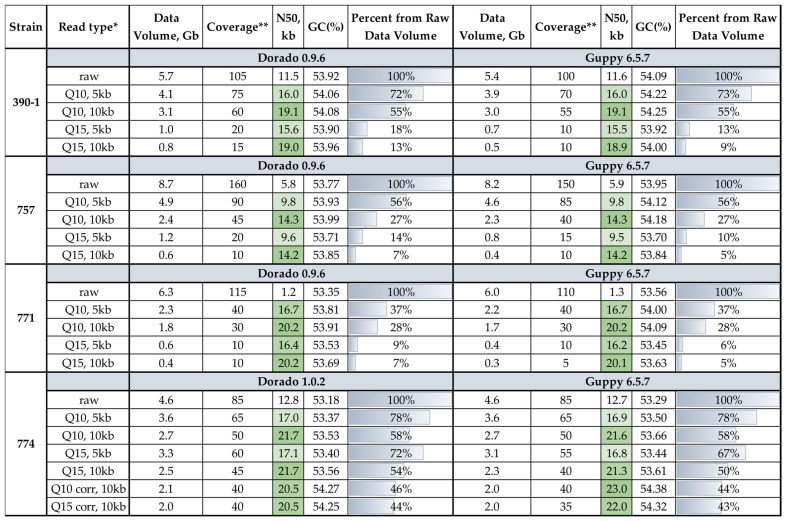
Statistics of the raw and filtered by quality (Q > 10 or Q > 15) and minimum read length (>5 kb or >10 kb) ONT data for *C. lini* strains #390-1, #757, and #771 (R9.4.1 data) and #774 (R10.4.1 data). * Read type: raw—basecalled and adapter trimmed with Porechop, Q10—with quality threshold Q = 10, Q15—with quality threshold Q = 15, 5 kb—with minimum read length of 5 kb, 10 kb—with minimum read length of 10 kb, corr—corrected with Dorado correct module. ** The estimated genome length is 55 Mb. The quality of the N50 value is indicated by the green (best)–white (worst) color scale. The percent of data volume from raw reads is indicated with gray–blue filling lines.

For the analysis of read N50 and genome coverage effects, we filtered reads by average quality (Q > 10 or Q > 15) and by length (length > 5 kb or length > 10 kb). In addition, we corrected reads obtained from the R10.4.1 flow cell (*C. lini* strain #774) using the built-in Dorado correct module. The statistics of the received reads were analyzed with SeqKit (Figure 1).

The data volume was between 0.3 and 8.7 Gb among all strains. The *C. lini* strain #774 genome coverage with R10.4.1 reads varied from 85× to 35×. The other strains’ genome coverage with R.9.4.1 reads varied from 160× to 5×. This difference was connected to the higher average read quality of R10.4.1 sequencing.

### 3.3. Impact of Read Processing on Assembly Quality

**Figure 2:**
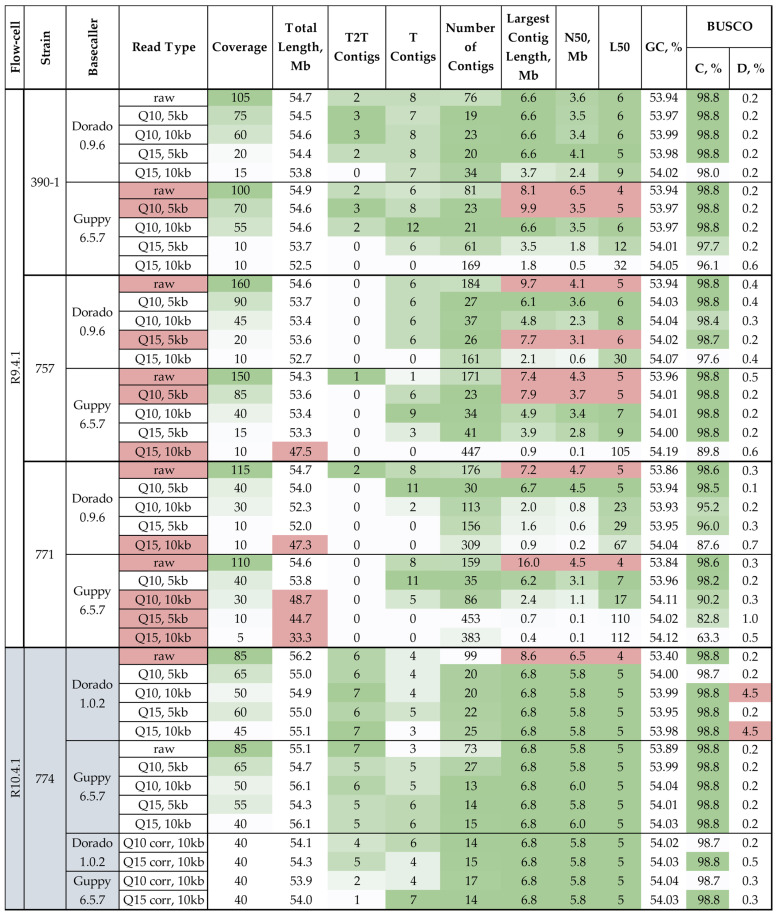
Statistics of genome assemblies produced by Hifiasm for *C. lini* strains #390-1, #757, #771, and #774 from ONT raw data and data with different filtration by quality (Q > 10 or Q > 15) and minimum read length (>5 kb or >10 kb). T2T contigs—number of telomere-to-telomere assembled contigs. T contigs—number of contigs with telomeric repeats on one end. BUSCO: C—complete, D—duplicated. The quality of the values is indicated by the green (best)–white–red (worst) color scale. The assemblies with a total length under 50 Mb (incomplete assembly) are indicated in red in the “Total Length” and “Read Type” columns. The assemblies with the largest contig length over 6.8 Mb (erroneously merged contigs) are indicated in red in the “Largest Contig Length”, “N50”, “L50”, and “Read Type” columns.

To assemble *C. lini* genomes, we used the Hifiasm tool. Using the generated combinations of ONT reads, we produced genome assemblies. The uncorrected reads were used with the --ont module, and the assemblies from the corrected *C. lini* strain #774 reads were obtained without any special modules. Hifiasm was developed to produce T2T genome assemblies, so one of the most significant assembly characteristics was the number of T2T-assembled chromosomes. Assembly quality was analyzed with QUAST and BUSCO. The presence of assembled telomeric repeats was verified with Tidk (Figure 2).

The estimated *C. lini* genome length is ~55 Mb. The *C. lini* genome contains ten core chromosomes (chromosome length > 0.9 Mb) and from two to four accessory chromosomes (chromosome length < 0.9 Mb) [23,32]. We checked the total length of all assemblies and marked those with atypical lengths (less than 50 Mb) in red in the “Total Length” and “Read Type” columns in Figure 2. The largest contig length was also checked, and the assemblies with the largest contig over 6.8 Mb (estimated length of the largest *C. lini* chromosome) were marked in red in the corresponding column and in the “Read type” column as the assemblies with merged chromosomes. We also indicated such assemblies in red in the “N50” and “L50” columns since the erroneously merged contigs affect these metrics.

There were five assemblies with a total length of less than 50 Mb; three of them were produced from Guppy-basecalled reads of strain #771, one from Dorado-basecalled reads of strain #771, and one from Guppy-basecalled reads of strain #757. All these assemblies were produced from R9.4.1 sequencing data. The majority of the assemblies with erroneously merged contigs (eight of nine) were also produced from R9.4.1 sequencing data.

The length of the genome assemblies obtained from raw reads was 54.3–56.2 Mb, and the largest contig length differed from 6.6 to 16.0 Mb, while the largest *C. lini* chromosome (Chromosome 1) is ~6.8 Mb long [23,32]. Only two raw read-based assemblies had no misassembled chromosomes—*C. lini* strain #390-1 genome assembly from Dorado-basecalled raw reads and *C. lini* strain #774 genome assembly from Guppy-basecalled raw reads. The raw read-based assemblies had from 76 to 184 contigs, despite the non-zero number of T and T2T contigs. This indicated that there were a significant number of small contigs in these assemblies. So, the Hifiasm assembler with unfiltered reads had a tendency to merge together two or more chromosomes while increasing the number of small contigs.

The genome assemblies of *C. lini* strain #774 from R10.4.1 sequencing data had no variety in the largest contig length (except for the assembly from raw Dorado-basecalled reads). For this strain, the N50 and BUSCO completeness values also had insignificant differences between the read types. The performance gap between R10.4.1 and R9.4.1 technologies was most apparent in the telomere resolution capability. Assemblies generated using R10.4.1 data had a substantially higher number of T2T contigs (5–7) compared to those based on R9.4.1 data (0–3).

GC content remained consistent across all assemblies (53.93–54.07%), indicating that neither sequencing technology nor read filtering introduced significant systematic bias in base composition. This consistency provides confidence in the biological validity of the assembled sequences.

The majority of the analyzed assemblies had more than 98.6% BUSCO completeness, which is comparable with that of the reference assemblies of the genus *Colletotrichum* from NCBI (https://www.ncbi.nlm.nih.gov/datasets/genome/?taxon=5455&reference_only=true, accessed on 12 September 2025). There was no correlation between the basecalling tool and the assembly completeness or percentage of missing genes. However, for three strains, the assemblies from Dorado-basecalled data had more assembled telomeres than the assemblies from Guppy-basecalled data.

For further analyses and for choosing the best (the most complete and contiguous) genome assembly for each strain, we took only assemblies from reads, which were not marked with red in Figure 2. In order to obtain the fairest and most unambiguous comparison, we performed the normalization of some characteristics for such assemblies using the following formula:

(1) $$X_{norm}=\frac{X-X_{min}}{X_{max}-X_{min}},$$

where *X* is the value to be normalized, *X_min_* is the minimum value of this characteristic among all analyzed assemblies, and *X_max_* is the maximum value of this characteristic among all analyzed assemblies.

**Figure 3:**
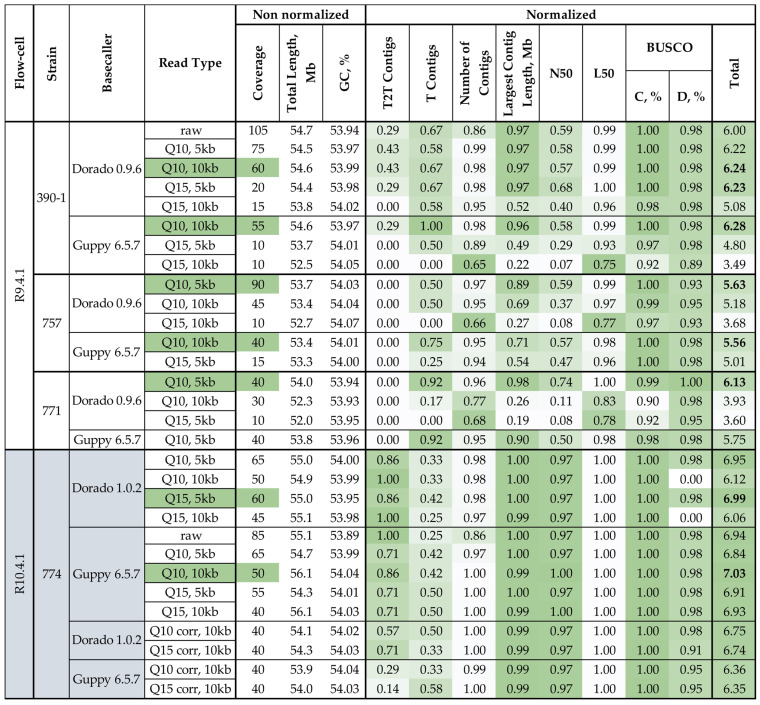
Normalized statistics for the genome assemblies of *C. lini* strains #390-1, #757, #771, and #774 produced by Hifiasm from ONT raw data and data with different filtration by quality (Q > 10 or Q > 15) and minimum read length (>5 kb or >10 kb). BUSCO: C—complete, D—duplicated. The quality of the values is indicated by the green (best)–white (worst) color scale. The “Total” is the sum of all normalized characteristics. Numbers in bold are the maximum “Total” values for each strain. The corresponding values in the “Read Type” and “Coverage” columns are colored green.

For normalization, we chose the number of contigs with both assembled telomeres (T2T contigs), the number of contigs with assembled telomeric repeats only at one end (T contigs) (with a 0.5 ratio), the number of contigs, the largest contig length, and N50, L50, and BUSCO (complete (C) and duplicated (D)) metrics. The total length and GC content are more qualitative than quantitative since there is no direct correlation between the assembly quality and the value of these metrics. So, we kept them non-normalized. The best assembly for every strain was chosen by the sum of normalized metrics (Figure 3).

The performed analysis revealed an optimal coverage for achieving high-quality *C. lini* genome assemblies. The moderate coverage (40–65×) consistently produced the best results. Excessively high coverage (>90×) did not necessarily improved the assembly quality. Coverage over 90× often led to the merging of chromosomes. We concluded that, when a certain threshold of coverage is surpassed, read quality and read length become more significant factors for successful assembly.

For R10.4.1 data specifically, coverage between 40× and 65× combined with Q10 quality filtration and 5–10 kb minimum read length filtration produced the highest-quality assemblies (total scores 6.84–7.03). The comparison of corrected and uncorrected R10.4.1 reads revealed that, in most cases, overall assembly quality was higher for assemblies obtained from uncorrected reads (total scores 6.35–6.75 for corrected data versus 6.06–7.03 for uncorrected data).

**Figure 4:**
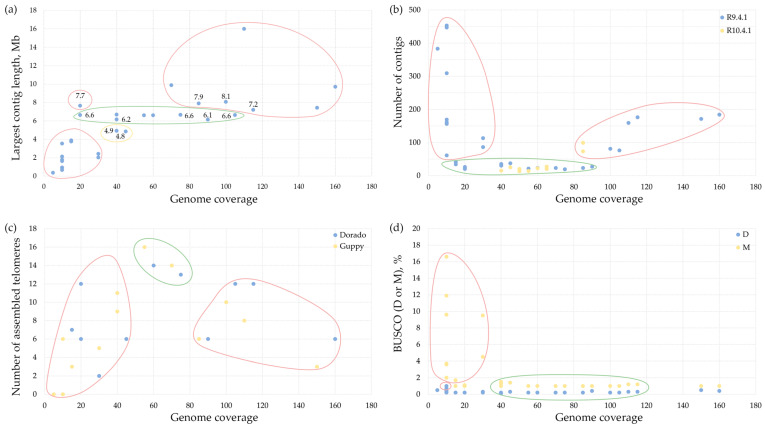
Relationship of genome coverage and (**a**) the largest contig length, (**b**) the number of contigs, (**c**) the number of assembled telomeres, (**d**) BUSCO (D—duplicated and M—missing) for the Hifiasm-produced genome assemblies from R9.4.1 data for *C. lini* strains #390-1, #757, and #771. The quality of the values is indicated by the green (best)–yellow–red (worst) color scale (see the color of shapes around the dots).

Filtration of R9.4.1 data for quality and read length demonstrated a complex relationship with assembly contiguity and completeness (Figure 4). The Q10 quality filtration and 5 kb or 10 kb minimum read length filtration generally improved assemblies’ contiguity by reducing the number of contigs (19–27 contigs for Q10-filtered assemblies versus 76–184 contigs for raw read-assemblies). However, excessive filtration with Q15 and a minimum read length of 10 kb was detrimental. The most significant assembly quality deterioration was observed when Q15 and 10 kb minimum read length filtration resulted in significant assembly fragmentation (169–447 contigs) (Figure 4b) and reduction in the N50 value (0.1–0.6 Mb), so T2T contigs completely disappeared (Figure 4c). Moreover, all assemblies from R9.4.1 data with the “Q15, 10 kb” read type had significantly lower BUSCO completeness and higher missing BUSCO values in comparison with other assemblies (Figure 4d). We believe that this was related to insufficient genome coverage with sequencing data. This pattern suggests that, while removing low-quality reads can benefit assembly, overly aggressive filtration eliminates reads essential for spanning repetitive regions and maintaining assembly continuity.

### 3.4. Comparison of the Obtained Genome Assemblies

**Figure 5:**
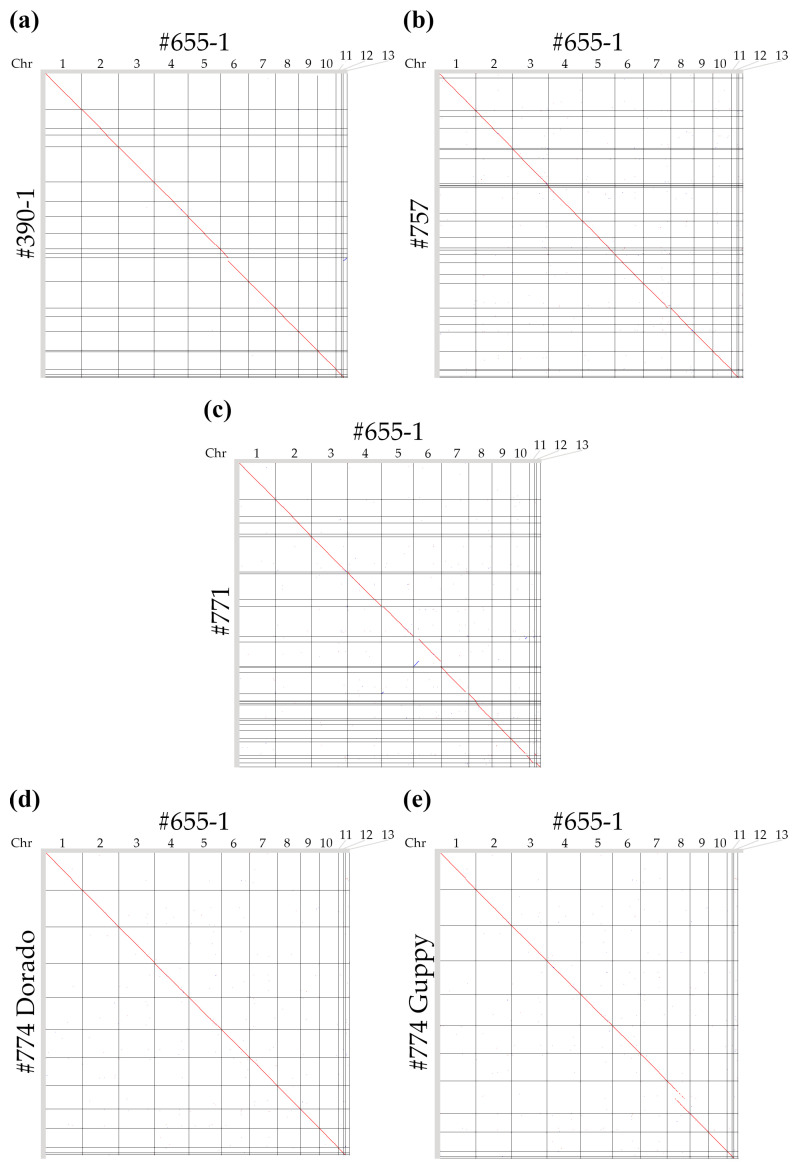
The results of alignment of genome assemblies for (**a**) *C. lini* strain #390-1 (Dorado-basecalled, “Q10, 10 kb” read type) and *C. lini* strain #655-1; (**b**) *C. lini* strain #757 (Dorado-basecalled, “Q10, 5 kb” read type) and *C. lini* strain #655-1; (**c**) *C. lini* strain #771 (Dorado-basecalled, “Q10, 5 kb” read type) and *C. lini* strain #655-1; (**d**) *C. lini* strain #774 (Dorado-basecalled, “Q15, 5 kb” read type) and *C. lini* strain #655-1; (**e**) *C. lini* strain #774 (Guppy-basecalled, “Q10, 10 kb” read type) and *C. lini* strain #655-1. Red lines indicate the forward orientation of the aligned sequences, and blue lines indicate the reverse orientation of the aligned sequences.

We made whole-genome alignments of the best obtained genome assemblies of four *C. lini* strains to the previously assembled complete genome of *C. lini* strain #655-1 (Figure 5) [23]. For R9.4.1-sequenced genomes (strains #390-1, #757, and #771), there were some fragmented chromosomes. *C. lini* strain #390-1 chromosomes 1, 3, 8, 9, 10, 11, and 12 were completely assembled (seven out of thirteen). *C. lini* strain #757 had four (out of twelve) completely assembled chromosomes (9, 10, 11, and 12) and one nearly complete chromosome (7), which might be a strain diversity. *C. lini* strain #771 had three (out of thirteen) completely assembled chromosomes (1, 3, 13) and some nearly complete chromosomes (5, 11, and 12). *C. lini* strain #774, sequenced using the R10.4.1 flow cell, had all twelve complete chromosomes in both assemblies. However, the assembly from Guppy-basecalled reads had repeats on chromosome 8, which were not present in other assemblies of this strain’s genome. Therefore, we concluded that these repeats were assembly errors.

The quality of the data from R10.4.1 flow cell allowed us to obtain a T2T-level assembly of *C. lini* genome with all complete chromosomes without gaps. Data from R9.4.1 flow cell, which had a lower quality, were less suitable for this purpose, but Hifiasm also produced assemblies of *C. lini* genomes that were close to the T2T level. The comparison of the obtained assemblies with the assemblies of *C. lini* genomes from our previous study [33] revealed that they were very similar. In our previous research, we generated *C. lini* genome assemblies using Canu and then polished them in four iterations with ONT and Illumina reads using various tools. This resulted in the assemblies of 26–32 contigs. These assemblies did not contain a single copy of the mitochondrial genome, and it was necessary to manually search for it and separate it. Fragmented contigs were also present. Considering the time and CPU resources used to assemble the *C. lini* genome, we found that Hifiasm was more efficient than Canu in terms of the time–quality ratio.

According to our previous research [23], the best Illumina polishing tool for Hifiasm-assembled *C. lini* genomes was Pilon. Thus, we performed polishing with Pilon for the obtained assemblies. The assemblies’ coverage with trimmed Illumina reads was 25–35× for each strain (the estimated genome length is 55 Mb). The percentage of mapped Illumina reads for each *C. lini* strain was more than 96% (#390-1—96.3%, #757—98.4%, #771—99.0%, #774—99.9%). We evaluated the polished assemblies’ completeness with BUSCO, and there were no changes compared to the unpolished assemblies. The number of mismatches and indels per 100 kbp relative to the reference genome decreased insignificantly (by ~2% and by ~0.5%, respectively). Thus, the obtained Hifiasm assemblies of *C. lini* genomes did not require polishing with Illumina reads.

As a result, we accepted the genome assemblies of *C. lini* strain #390-1 from Dorado-basecalled reads with quality filtration Q10 and minimum read length of 10 kb, *C. lini* strain #757 from Dorado-basecalled reads with quality filtration Q10 and minimum read length of 5 kb, *C. lini* strain #771 from Dorado-basecalled reads with quality filtration Q10 and minimum read length of 5 kb, and *C. lini* strain #774 from Dorado-basecalled reads with quality filtration Q15 and minimum read length of 5 kb as the final assemblies (Table 2). For the *C. lini* strain #774, we filtered small contigs, as all chromosomes were already fully assembled.

## 4. Discussion

The most commonly used strategy for obtaining T2T genome assemblies currently involves the use of PacBio high-fidelity (HiFi) and ultra-long ONT reads, as well as Hi-C data [44,45,46]. Popular assemblers for ONT reads include Canu and Hifiasm [18,47,48]. In some instances, researchers used Canu to generate separate assemblies from PacBio HiFi and ONT reads and then combined them using Hifiasm [49]. For an assembly from only ONT reads, Canu was earlier the primary assembler [31,32,50,51,52,53]. For Hifiasm, it was necessary to preprocess ONT reads using the Dorado correct module (HERRO) for single-read error correction (https://github.com/nanoporetech/dorado, accessed on 12 September 2025), which was suitable only for the R10.4.1 data [35]. The performance of Hifiasm, when used with the --ont module, demonstrated a significant improvement in the assembly of *C. lini* genomes. The opportunity to generate near-T2T assemblies using ONT simplex reads from R9.4.1 and R10.4.1 flow cells, without the need for read correction or Illumina polishing, greatly simplifies the assembly process while maintaining high-quality results. Our comparison with previous Canu-produced *C. lini* genome assemblies [33] showed that Hifiasm achieved similar or better results with substantially reduced computational requirements and without the need for multiple polishing iterations.

The comparison between the Dorado and Guppy basecallers revealed important differences in read quality assessment. While both basecallers produced usable data, Dorado consistently generated reads with higher average quality scores, particularly for R10.4.1 data (19.2 vs. 17.1). Comparative analyses of basecallers are regularly conducted. Several years ago, before the Dorado development, the Guppy basecaller was considered the best one [54]. In modern research, Dorado is recognized as the best ONT basecaller [43]. This difference in read quality proved significant for downstream assembly quality, as assemblies from Dorado-basecalled data consistently showed better telomere resolution. The consistent recovery of complete mitochondrial genomes in assemblies from Dorado-basecalled data is another important finding. The presence of fully assembled mitochondrial genomes in a single copy suggests superior assembly continuity and completeness. This has implications for evolutionary studies and population genetics, as mitochondrial genomes provide valuable phylogenetic information [55,56,57].

Our analysis of coverage requirements revealed that moderate coverage (40–65×) consistently produced optimal results, while excessively high coverage (>90×) did not necessarily improve assembly quality and sometimes led to chromosome merging artifacts. A similar study was conducted for Hifiasm when working with PacBio HiFi data, and the researchers obtained a similar range of genome coverage levels, which helped to create the best assemblies in terms of contiguity and completeness [58]. This finding challenges the conventional inference that higher coverage always improves assembly statistics and suggests that beyond a certain threshold, read quality and length become more critical factors than the coverage itself. This has practical implications for sequencing experiment design, potentially reducing the cost and time requirements for generating high-quality fungal genome assemblies.

While our study focused specifically on *C. lini*, the methodologies and insights gained have broader applicability to other fungi with comparable genome lengths. Despite the existing diversity, they share general structural characteristics and present similar challenges during the assembly process. The principles of choosing the optimal coverage, read filtration thresholds, and assembly algorithm can be applied in genome assembly projects for other fungi. This is particularly relevant for species with genome architectures similar to *Colletotrichum*, including other plant pathogens and medically important fungi [59,60].

## 5. Conclusions

The performed research identified that the ONT R10.4.1 flow cell and Dorado-basecalled data with a moderate genome coverage (40–65×) and thresholds of a Q15 average read quality and 5 kb minimum read length was the optimal configuration for achieving complete, contiguous, and accurate assemblies of *C. lini* genomes using Hifiasm with the --ont module. For the *C. lini* genome assembly from the ONT R9.4.1 data, we could recommend Dorado-basecalled reads with Q10 quality and 5 kb minimum read length thresholds and genome coverage of 40–90×.

These findings have important implications for fungal genome assembly projects, particularly for applications requiring complete T2T assemblies to resolve complex genomic regions.

## Figures and Tables

**Table 1:** Statistics of the basecalled ONT genome sequencing data after adapter trimming for *C. lini* strains #390-1, #757, and #771 (R9.4.1 data) and #774 (R10.4.1 data).

Strain	Basecaller	Data Volume, Gb	Genome Coverage ^1^, ×	Max Read Length, kb	N50, kb	Percentage of Data with Q > 20, %	Average Read Q	GC, %
390-1	Dorado 0.9.6	5.7	105	104.8	11.5	60.1	13.3	53.92
	Guppy 6.5.7	5.4	100	377.4	11.6	59.2	13.1	54.09
757	Dorado 0.9.6	8.7	160	147.5	5.8	59.9	13.3	53.77
	Guppy 6.5.7	8.2	150	115.7	5.9	59.1	13.0	53.95
771	Dorado 0.9.6	6.3	115	116.2	1.2	60.1	13.4	53.35
	Guppy 6.5.7	6.0	110	219.7	1.3	59.6	13.2	53.56
774	Dorado 1.0.2	4.6	85	152.2	12.8	91.8	19.2	53.18
	Guppy 6.5.7	4.6	85	150.5	12.7	82.1	17.1	53.29

^1^ The estimated genome length is 55 Mb.

**Table 2:** Statistics of the final genome assemblies produced by Hifiasm (--ont) from Dorado-basecalled reads for *C. lini* strains #390-1, #757, #771, and #774.

Strain	Read Type	Coverage ^1^, ×	Assembly Length, Mb	Number of Contigs	Number of Chromosomes	Number of Complete Chromosomes ^2^	N50, Mb	L50	GC, %	BUSCO ^3^	
										C, %	D, %
390-1	Q10, 10 kb	60	54.6	23	13	7	3.4	6	53.99	98.8	0.2
757	Q10, 5 kb	90	53.7	27	12	4	3.6	6	54.03	98.8	0.4
771	Q10, 5 kb	40	54.0	30	13	3	4.5	5	53.94	98.5	0.1
774	Q15, 5 kb	60	54.4	13	12	12	5.8	5	53.95	98.8	0.2

^1^ The estimated genome length is 55 Mb. ^2^ Complete chromosomes—fully assembled chromosomes (might be without telomeric repeats). ^3^ BUSCO: C—complete, D—duplicated.

## Data Availability

The data generated in this study can be found in the NCBI database under the BioProject PRJNA929545 (linked to BioSample SAMN13621712).
